# Three-Dimensional Heart Model-Based Screening of Proarrhythmic Potential by *in silico* Simulation of Action Potential and Electrocardiograms

**DOI:** 10.3389/fphys.2019.01139

**Published:** 2019-09-04

**Authors:** Minki Hwang, Seunghoon Han, Min Cheol Park, Chae Hun Leem, Eun Bo Shim, Dong-Seok Yim

**Affiliations:** ^1^SiliconSapiens Inc., Seoul, South Korea; ^2^Department of Clinical Pharmacology and Therapeutics, Seoul St. Mary’s Hospital, Seoul, South Korea; ^3^Pharmacometrics Institute for Practical Education and Training (PIPET), College of Medicine, The Catholic University of Korea, Seoul, South Korea; ^4^Department of Mechanical and Biomedical Engineering, Kangwon National University, Chuncheon, South Korea; ^5^Department of Physiology, College of Medicine, University of Ulsan, Asan Medical Center, Seoul, South Korea

**Keywords:** 3D heart model, ECG simulation, hERG, QT, torsade de pointes

## Abstract

The proarrhythmic risk is a major concern in drug development. The Comprehensive *in vitro* Proarrhythmia Assay (CiPA) initiative has proposed the JTpeak interval on electrocardiograms (ECGs) and qNet, an *in silico* metric, as new biomarkers that may overcome the limitations of the hERG assay and QT interval. In this study, we simulated body-surface ECGs from patch-clamp data using realistic models of the ventricles and torso to explore their suitability as new *in silico* biomarkers for cardiac safety. We tested seven drugs in this study: dofetilide (high proarrhythmic risk), ranolazine, verapamil (QT increasing, but safe), bepridil, cisapride, mexiletine, and diltiazem. Human ventricular geometry was reconstructed from computed tomography (CT) images, and a Purkinje fiber network was mapped onto the endocardial surface. The electrical wave propagation in the ventricles was obtained by solving a reaction-diffusion equation using finite-element methods. The body-surface ECG data were calculated using a torso model that included the ventricles. The effects of the drugs were incorporated in the model by partly blocking the appropriate ion channels. The effects of the drugs on single-cell action potential (AP) were examined first, and three-dimensional (3D) body-surface ECG simulations were performed at free Cmax values of 1×, 5×, and 10×. In the single-cell and ECG simulations at 5× Cmax, dofetilide, but not verapamil or ranolazine, caused arrhythmia. However, the non-increasing JTpeak caused by verapamil and ranolazine that has been observed in humans was not reproduced in our simulation. Our results demonstrate the potential of 3D body-surface ECG simulation as a biomarker for evaluation of the proarrhythmic risk of candidate drugs.

## Introduction

The proarrhythmic effects of cardiac and non-cardiac drugs have comprised a major drug safety issue for the past 20 years (Zipes, 1987; De Ponti et al., 2002; Moro et al., 2010). The electrocardiogram (ECG) is an effective means of determining whether a drug is proarrhythmic. Under certain conditions, prolongation of the QT interval increases the risk of developing Torsades de pointes (TdP), which can lead to sudden cardiac death (Thomas and Behr, 2016). The Comprehensive *in vitro* Proarrhythmia Assay (CiPA) was recently proposed to improve the accuracy of drug safety prediction during preclinical and clinical development (Vicente et al., 2018; Wallis et al., 2018). The CiPA comprises *in silico* simulation of several ion-channel assays and ECG studies to identify biomarkers of false-positive results of single hERG channel assays and thorough QT (TQT) studies (Darpo, 2010) performed according to the International Council for Harmonisation (ICH) S7B and E14 guidelines. There have been a large number of studies that investigated the effect of drugs on ECG using in silicon three-dimensional (3D) heart model. Zemzemi et al. (2013) examined the effect of the block of ion channels on ECG parameters. Okada et al. (2018) generated an arrhythmic hazard map under multiple ion channel blocks. Sahli Costabal et al. (2019) investigated the critical drug concentration which induced torsade de pointes. Rivolta et al. (2017) performed sensitivity analysis of JT_peak_ and T-wave morphology parameters.

In this study, we further examined the utility of 3D ECG simulation in evaluating drug safety by simulating ECG at relatively high concentrations of drugs using realistic models of the ventricles and torso. We tested dofetilide, bepridil, cisapride, ranolazine, verapamil, mexiletine, and diltiazem using the 3D model and examined the morphologies of the simulated ECG data according to drug concentration. Dofetilide, bepridil, and cisapride are high or intermediate-proarrhythmic-risk drug that prolongs the QT by blocking hERG. Verapamil and ranolazine are “false positive” low-proarrhythmic-risk drugs; they induce prolonged QT by hERG blockade while simultaneously blocking inward Ca^2+^ (verapamil) and Na^+^ (ranolazine) ion channels (Vicente et al., 2018). Mexiletine and diltiazem are low-proarrhythmic-risk drugs that do not prolong the QT at all. Recently, CiPA researchers proposed a new ECG biomarker, JTpeak, which may enable the identification of drugs producing false-positive results (Vicente et al., 2018). Proarrhythmic drugs prolong the QT and the JTpeak due to hERG blockade, but not when the hERG blockade is offset by simultaneous blockade of other depolarizing ion channels (as by verapamil and ranolazine: only the QT prolonged but not the JTpeak). In this study, we explored the ability of the results of 3D ECG simulations to identify false-positive results independently of clinically obtained ECG data.

## Materials and Methods

### ECG Simulation Using Models of the Ventricles and Torso

**FIGURE 1:**
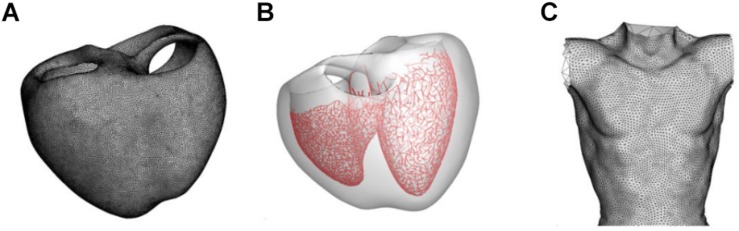
Models of the ventricle and torso used in the present study. **(A)** Grid of the ventricular model. **(B)** Model of the Purkinje fiber network. **(C)** Model of the torso.

The model construction and ECG simulation are also described in our previous papers (Im et al., 2008; Lim et al., 2013; Ryu et al., 2019). Human ventricular geometry and torso were from our previous studies (Lim et al., 2013; Ryu et al., 2019) (Figures 1A–C). Human ventricular geometry was reconstructed from the computed tomography (CT) images obtained from the University of Ulsan Medical Center using a commercially available software Aquarius intuition (TeraRecon Inc., San Mateo, CA, United States). Tetrahedral mesh was generated inside the 3D ventricular model using an in-house software (Figure 1A). The number of grid element was 1,475,818. For the modeling of Purkinje fibers, the 2-dimensional representation of the Purkinje network shown in the paper by Berenfeld and Jalife (1998) was digitized, scaled to the size of the 3D model, and mapped onto the endocardial surface of the 3D model of the ventricles (Figure 1B). Pacing was applied at the location of His bundle. The model of Purkinje fibers simply transmits the electrical signal unidirectionally. The speeds of signal transmission at various regions were adjusted manually so that the simulated activation map matches that of clinical data (Durrer et al., 1970; Supplementary Figure S2). The end nodes of the Purkinje network stimulated myocardium by applying stimulation current of −80.0 A/F until the membrane potential exceeds −10 mV. The endocardial nodes connected to the node nearest to each end node of the Purkinje network were considered the Purkinje-muscle junction (PMJ). All the tetrahedral elements containing the PMJ nodes were stimulated. Signal propagation from the stimulation nodes throughout the tissue was obtained by solving a reaction-diffusion equation (Eq. 1) numerically:

(1) $$\frac{\partial V_{\text{m}}}{\partial t}=\nabla \cdot D\,\nabla V_{\text{m}}-\left(I_{\text{ion}}+I_{\text{stim}}\right)\,\frac{1}{C_{\text{m}}},$$

where *V*_m_ is the transmembrane potential, *t* is time, *D* is the diffusion tensor, *C*_m_ is the membrane capacitance, and *I*_ion_ and *I*_*stim*_ are ionic and stimulation currents, respectively. The equation was spatially discretized by using finite element and the time derivative was approximated by forward Euler method (Im et al., 2008). To calculate *I*_ion_, the O’Hara-Rudy dynamic (ORd) human ventricular cell model was used (O’Hara et al., 2011). ORd model has three types of cells: endocardial, M, and epicardial cells. Each cell type was assigned to the ventricular wall with reference to the figure shown in the paper by Trudel et al. (2004). We also tested three recently published optimized cell models (Mann et al., 2016; Dutta et al., 2017; Krogh-Madsen et al., 2017). To calculate ECG values, the boundary element model of the human torso proposed by Potse et al. (2009) was used (Figure 1C). The ECG was calculated by computing the potentials on the torso surface using the following equation (Potse et al., 2009):

(2) $$\phi_{e\,k}\,\left(r\right)=\frac{1}{2\,\pi \,\left(\sigma_{k}^{-}+\sigma_{k}^{+}\right)}\cdot \left[\int J_{c}\,\left(r^{\prime }\right)\cdot \frac{r-r^{,3}}{{\left|r-r^{\prime }\right|}^{3}}\,\text{d}\,V^{\prime }+\sum_{l}\int_{S\,l}\left(\sigma_{l}^{-}-\sigma_{l}^{+}\right)\,\phi_{e}\,\left(r^{\prime \prime }\right)\,\text{d}\,\Omega_{r\,r^{\prime \prime }}\right]$$

where ϕ_*ek*_(*r*) is potential at a point r on surface k. $\sigma_{k}^{-}$ and $\sigma_{k}^{+}$ are the conductivity inside and outside the surface k, respectively, *J*_*c*_ is the source current density field, and *r*′ and *r*″ are variables. The summation is over all surfaces *l*. dΩ_*rr*″_ is the solid angle subtended at *r* by the infinitesimal surface element located at *r*″. The key parameters of simulation are shown in Table 1.

**TABLE 1:** Key parameters of simulation.

Number of computational elements	1,475,818
Ventricular tissue diffusion coefficient	0.00154 cm^2^/s
Ventricular cell membrane capacitance	2.0 μF/cm^2^
Body conductivity	2.0 mS/cm

### Incorporation of the Effects of Drugs in the ECG Simulation

For the simulation of drug effect on ECG, we used the parameter values obtained by CiPA researchers to compare with their clinical data (Crumb et al., 2016; Li et al., 2017, 2019). We tested seven drugs: dofetilide, bepridil, cisapride, verapamil, ranolazine, mexiletine, and diltiazem. The effects of drugs were incorporated in the ECG simulation by partly blocking the corresponding ion channels (*I*_Na_, *I*_NaL_, *I*_CaL_, and *I*_Kr_) in the ionic-current model. The percentage of blockage of each ionic current was calculated using the Hill equation (Goutelle et al., 2008). The Cmax, IC_50_, and Hill coefficient values for each drug with respect to each ionic current were adopted from the literature (Table 2) (Crumb et al., 2016; Li et al., 2017, 2019). Cmax means free Cmax unless otherwise stated. The effects of each drug on single-cell action potentials [of endocardial (endo), epicardial (epi), and mid-myocardial (M) cells] were examined first, and 3D ECG simulations were performed at 1×, 5× and 10× Cmax.

**TABLE 2:** Percentages of blockage of four ionic currents.

	***I*_NaL_**	***I*_CaL_**	***I*_Na_**	***I*_Kr_(hERG)**
**Dofetilide**				
Cmax (μM)	0.002	0.002	0.002	0.002
IC50 (μM)	126	44.5	1.36	0.001
Hill coefficient	1.1	3.6	1.1	0.6
Block at Cmax (%)	0.000526	2.24E-14	0.0765	60.2
**Bepridil**				
Cmax (μM)	0.033	0.033	0.033	0.033
IC50 (μM)	1.82	2.82	2.96	0.149
Hill coefficient	1.4	0.65	1.2	0.9
Block at Cmax (%)	0.363	5.26	0.452	20.5
**Cisapride**				
Cmax (μM)	0.0026	0.0026	0.0026	0.0026
IC50 (μM)	9260	1030	1790	0.012
Hill coefficient	6.3	4.8	0.67	1.3
Block at Cmax (%)	5.3E-40	1.35E-25	0.0123	12.0
**Verapamil**				
Cmax (μM)	0.081	0.081	0.081	0.081
IC50 (μM)	24.1	0.204	2590	0.499
Hill coefficient	2	1.1	3.5	1.1
Block at Cmax (%)	0.00113	26.6	1.71E-14	11.9
**Ranolazine**				
Cmax (μM)	1.95	1.95	1.95	1.95
IC50 (μM)	7.94	900	53.3	6.49
Hill coefficient	0.95	3.9	1.9	0.8
Block at Cmax (%)	20.8	4.06E-9	0.186	27.6
**Mexiletine**				
Cmax (μM)	4.13	4.13	4.13	4.13
IC50 (μM)	9.02	38.9	26.1	Infinity
Hill coefficient	1.4	1	3.8	−
Block at Cmax (%)	25.1	9.60	0.0905	0
**Diltiazem**				
Cmax (μM)	0.122	0.122	0.122	0.122
IC50 (μM)	21.6	0.113	36.9	6.57
Hill coefficient	0.68	0.72	1.4	0.8
Block at Cmax (%)	2.87	51.4	0.0336	3.96

*The percentages were calculated using the Hill equation applying the Cmax, IC50, and Hill coefficient values adopted from the literatgure (Crumb et al., 2016; Li et al., 2017, 2019). Cmax (free Cmax) values are from Li et al. (2017). IC_50_ and Hill coefficient values of hERG are from Crumb et al. (2016). IC_50_ and Hill coefficient values of Ca^2+^ and Na^+^ are from Li et al. (2019).*

## Results

### Effects on Single-Cell APs

**FIGURE 2:**
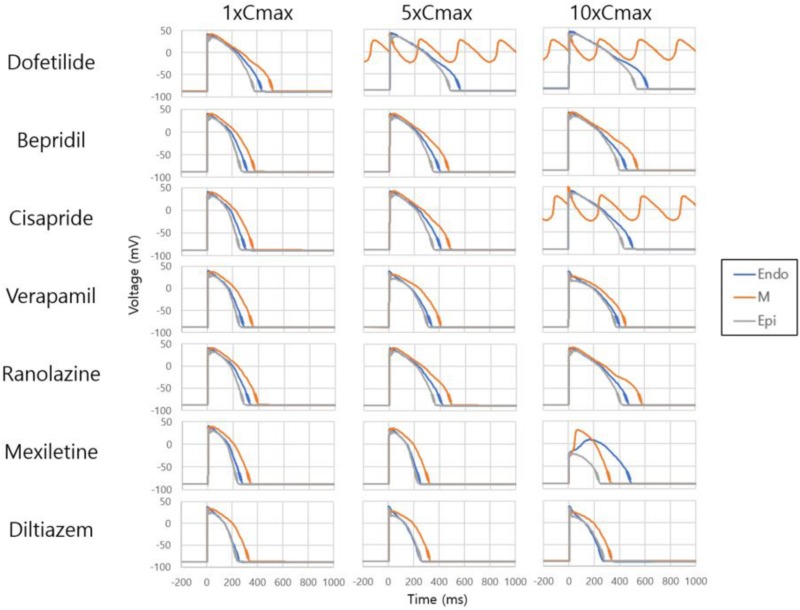
Effects on single-cell action potential. Action potential curves are shown for 7 drugs at Cmax values of 1×, 5×, and 10×. The action potentials of endocardial, M, and epicardial cells are shown for each drug and Cmax value. Dofetilide at Cmax values of 5× and 10× and cisapride at 10× induced tachycardia.

**FIGURE 3:**
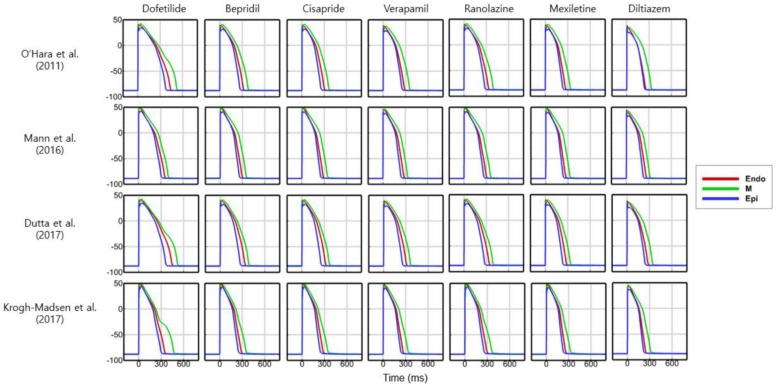
Action potentials with different cell models. Action potential curves are shown for 7 drugs with 4 different cell models at 1× Cmax. Action potentials of endocardial, M, and epicardial cells are shown.

Figure 2 shows AP curves for endocardial, M, and epicardial cells for seven drugs at Cmax values of 1×, 5×, and 10× (Supplementary Table S1). The Cmax values are listed in Table 2. The increases in the 90% AP duration (APD_90_) for the three drugs in endocardial, M, and epicardial cells compared with the no-drug control are shown in Table 3. Among the three drugs, dofetilide induced the greatest increase in the APD_90_ value, followed by ranolazine and verapamil. When Cmax was increased from 1× to 10×, APD_90_ increased for all three drugs and all three cell types, with the exception of M cells, in the presence of dofetilide. Dofetilide induced ventricular tachycardia in the M cells at Cmax values of 5× and 10× (Figure 2). Table 4 shows the transmural dispersion of repolarization (TDR) values, calculated as the difference between the largest and smallest APD_90_s among the endocardial, M, and epicardial cells. The TDR was largest in the case of dofetilide, and verapamil did not alter the TDR at a Cmax of 1× compared with the drug-free control (Table 4). At a Cmax of 10×, verapamil increased the APD_90_ of epicardial cells to a greater degree than that of M cells (Table 3), which resulted in a decreased TDR compared with the drug-free control (Table 4). Figure 3 shows AP curves for seven drugs with different cell electrophysiology models. The models of O’Hara et al. (2011) and Dutta et al. (2017) provided relatively long ADP. Safe drugs resulted in relatively short APD except for ranolazine in which metabolites seem to play a significant role in drug binding (Moreno et al., 2013).

**TABLE 3:** Simulated ΔAPD_90_ for endocardial, M, and epicardial cells (Units: ms).

**Drugs**	**1× Cmax**			**5× Cmax**			**10× Cmax**		
	**Endo**	**M**	**Epi**	**Endo**	**M**	**Epi**	**Endo**	**M**	**Epi**
Dofetilide	157	182	136	277	–	239	341	–	288
Bepridil	34	37	28	115	127	104	174	201	158
Cisapride	21	21	17	124	138	108	225	–	194
Verapamil	4	12	13	57	71	69	123	113	135
Ranolazine	49	50	43	131	146	120	188	235	170
Mexiletine	–13	–11	–7	–36	–29	–12	209	–14	5
Diltiazem	–30	–14	–2	–29	–16	15	–18	–6	34

**TABLE 4:** Simulated changes in TDR, QTc, and JT_peak_c according to drug concentration (Unit: ms).

**Drugs**	**1× Cmax**			**5× Cmax**			**10× Cmax**		
	**ΔTDR**	**ΔQTc**	**ΔJT_peak_c**	**ΔTDR**	**ΔQTc**	**ΔJT_peak_c**	**ΔTDR**	**ΔQTc**	**ΔJT_peak_c**
Dofetilide	46	266	247	–	–	–	–	–	–
Bepridil	9	51	44	23	179	179	43	289	281
Cisapride	4	30	26	31	194	191	–	–	–
Verapamil	0	17	18	2	79	83	–23	137	166
Ranolazine	7	70	74	26	216	206	64	–	–
Mexiletine	–4	–10	–9	–17	–	–	142	–	–
Diltiazem	–12	–19	–20	–27	–30	–1	–27	–9	24

### Effects on 3D ECG Parameters

**FIGURE 4:**
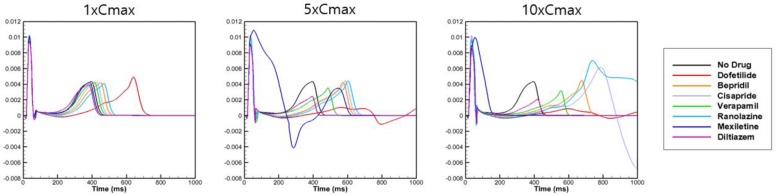
Effects on body-surface ECG parameters. Body-surface ECG data (lead I) are shown for 7 drugs at Cmax values of 1×, 5×, and 10× compared with drug-free conditions. The QT interval was longest for dofetilide.

**FIGURE 5:**
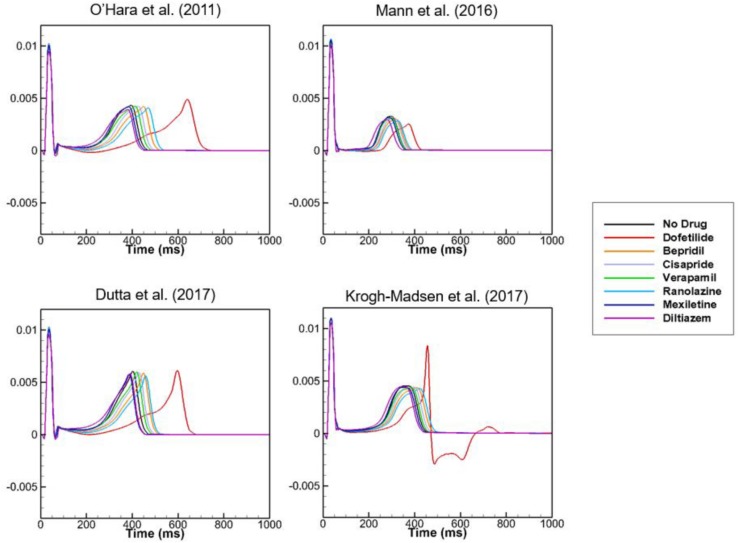
ECGs with different cell models. Body-surface ECGs (lead I) are shown for 7 drugs applying different cell models at 1× Cmax. Irregular morphology of ECG is observed for dofetilide in the case of the model by Krogh-Madsen et al. (2017).

To examine the effects of drug concentration on ECG parameters, 3D ECG simulations were performed using the conditions shown in Figure 2. Figure 4 shows the simulated ECGs (lead I) for the seven drugs according to concentration. Dofetilide resulted in the greatest increase in the ΔQTc value at 1× Cmax (Table 4). At a Cmax value of 5×, dofetilide induced ventricular flutter; at 10× Cmax, dofetilide, cisapride, and ranolazine induced ventricular flutter. In contrast to findings reported by the CiPA researchers (Vicente et al., 2018), the JT_peakC_ value increased with the QTc value for ranolazine and verapamil (Table 4). Figure 5 shows ECGs obtained from using different optimized cell models for the seven drugs at 1× Cmax. Dofetilide exhibited relatively long QT interval in all the cell models except for the model of Krogh-Madsen et al. (2017) in which the ECG morphology was irregular. The amplitude of the T wave was largest in the case of Dutta et al. (2017) while the model of Mann et al. (2016) exhibited the smallest T wave amplitude. Table 5 shows JT_peak_c prolongation of drugs for different optimized cell models. The optimized cell models resulted in JT_peak_c prolongations which are more consistent with clinical observations than the original ORd model. For ranolazine, metabolites seem to play a significant role in drug binding (Moreno et al., 2013).

**TABLE 5:** Simulated ΔJT_peak_c for various drugs with different cell models (Unit: ms).

**Drugs**	**O’Hara et al. (2011)**	**Mann et al. (2016)**	**Dutta et al. (2017)**	**Krogh-Madsen et al. (2017)**
Dofetilide	247	67	142	127
Bepridil	44	13	35	49
Cisapride	26	7	21	8
Verapamil	18	1	12	5
Ranolazine	74	32	43	58
Mexiletine	–9	–5	–14	–6
Diltiazem	–20	–15	–16	–4

**FIGURE 6:**
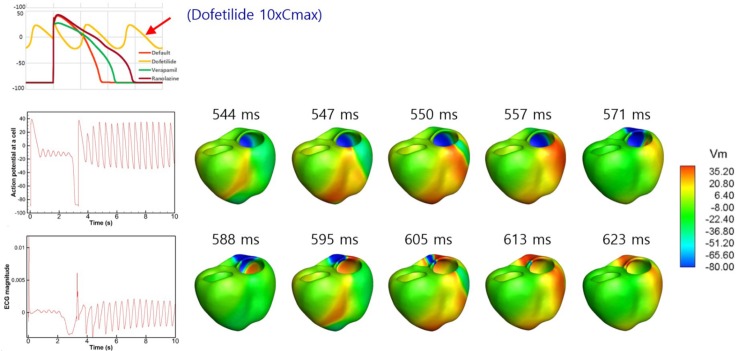
Drug-induced ventricular tachycardia. The action potential from the single-cell model, action potential at a point in the 3D model, and ECG data from the 3D model in the presence of dofetilide at a Cmax value of 10× are shown. Photographs of ventricular action potential propagation are also shown.

Because dofetilide induced ventricular tachycardia in the single-cell model at Cmax values of 5× and 10×, ventricular tachycardia was examined in the 3D model. Figure 6 shows the AP from the single-cell model, the AP at a point in the 3D model, and ECG data from the 3D model in the presence of dofetilide at a Cmax of 10×, which indicates the presence of ventricular tachycardia. Figure 6 also shows snapshots of ventricular AP propagation, which exhibits rotational activation. Table 6 lists the occurrences of ventricular tachycardia caused by the three drugs according to concentration. The arrhythmia morphology was not polymorphic, which is a limitation of our model.

**TABLE 6:** Occurrence of ventricular tachycardia. Simulated ECG was examined to determine the occurrence of VT (O: VT occurred, X: VT did not occur).

	**1× Cmax**	**5× Cmax**	**10× Cmax**
Dofetilide	X	O	O
Bepridil	X	X	X
Cisapride	X	X	O
Verapamil	X	X	X
Ranolazine	X	X	O
Mexiletine	X	X	X
Diltiazem	X	X	X

**FIGURE 7:**
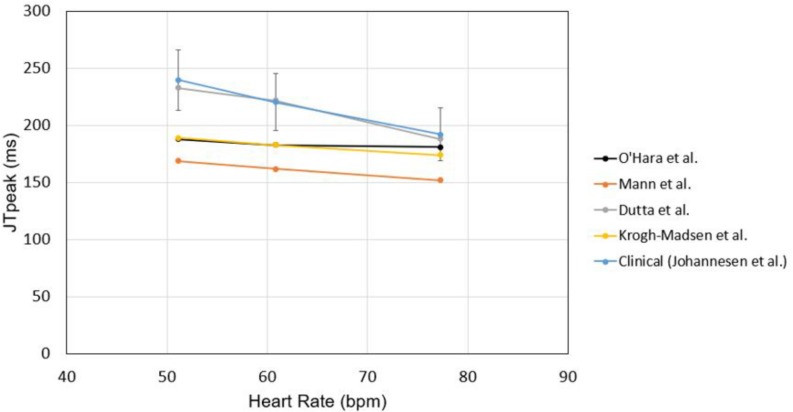
Rate dependence of JTpeak. JTpeak was obtained for different heart rates with different cell models. Simulation results were compared with clinical data by Johannesen et al. (2014).

To test the suitability of the models to examine JT_peak_, we checked the rate dependence of JT_peak_ using various models without any drug effect. All the models showed decreasing JT_peak_ as heart rate increased with the model of Dutta et al. (2017) exhibiting the best agreement with clinical data (Johannesen et al., 2014; Figure 7). We also validated intercellular conduction by comparing activation times obtained from our ventricular model with those in the literature (Supplementary Figure S1).

## Discussion

In evaluations of drug safety, the QT interval in ECGs has received much attention because drug-induced prolongation of the QT interval is, under certain conditions, associated with the risk of TdP, a fatal ventricular arrhythmia. However, prolongation of the QT interval does not always lead to TdP. The recently proposed CiPA initiative aims to enable more comprehensive evaluation of drug safety (Vicente et al., 2018; Wallis et al., 2018). In this study, we examined the effects of seven drugs on the QT interval using a realistic *in silico* 3D body-surface ECG model that included the ventricles and torso. Among the seven drugs, dofetilide resulted in the greatest increase in the QT interval, which is consistent with published data (Vicente et al., 2015). Dofetilide use entails a relatively high risk of TdP (Tisdale, 2016). However, the occurrence of TdP requires not only prolongation of the QT interval, but also early afterdepolarization (EAD) and TDR (Antzelevitch et al., 2004). In this study, dofetilide also exhibited the highest TDR values (Figure 2 and Table 4). In addition, dofetilide does not block the *I*_NaL_ and *I*_CaL_ channels (Table 2), which increases the probability of EAD. The increases in APD_90_ caused by ranolazine and verapamil were smaller than those induced by dofetilide, which resulted in smaller increases in the QT interval in the 3D ECG simulation. Ranolazine blocks *I*_NaL_ channels almost as effectively as *I*_Kr_ channels (Table 2) and entails a low risk of TdP because blockade of *I*_NaL_ channels decreases the risk of EAD (Hawwa and Menon, 2013). Interestingly, for ranolazine, the magnitude of the increase in the APD_90_ was similar in endocardial and M cells, whereas for verapamil it was similar in M and epicardial cells, at 1× Cmax (Table 3). Dofetilide induced the greatest increase in the APD_90_ of M cells; most QT interval-prolonging drugs increase the APD of M cells preferentially, thereby increasing the TDR value (Antzelevitch et al., 2004). Verapamil did not affect the TDR at a Cmax of 1× and decreased it at 10× Cmax, consistent with the low risk of TdP associated with its use (Milberg et al., 2005).

In this study, the increases in JT_peakC_ were similar to those in QTc, in contrast to the finding of Vicente et al. (2015) that the JT_peakC_ is not increased by ranolazine or verapamil at a Cmax value of 1×. T_peak_ corresponds to the time of epicardial repolarization, and most drugs that increase the epicardial APD also increase the JT_peakC_ because of delayed epicardial layer repolarization. Ranolazine and verapamil increased the epicardial APD and the JT_peakC_ in our simulation. Thus, the discrepancy in the JT_peakC_ interval between our simulated results and human ECGs performed after administration of verapamil or ranolazine implies that the 3D model needs further improvement. In order to make the model accurately predict the prolongations of JT_peak_ and T_peak_–T_end_, the improvement of the cell models seems to be needed. If epicardial APD remains the same, and endocardial APD increases under the effects of a drug, JTpeak should remain the same, and T_peak_–T_end_ should increase, which is expected in the cases of safe drugs. The current cell models do not exhibit these behaviors of APD changes under the effects of safe drugs, which disqualifies the current model as a biomarker. EAD was also observed in our simulated AP for ranolazine at a Cmax value of 10×, but not 5×. This result may reflect a limitation of the *in vitro* data-based simulation, in which the role of metabolites was not considered. However, simulation at a Cmax value of 10× was not recommended by the CiPA because of excessive variability in the values of the markers at higher concentrations (Li et al., 2019). Thus, the ventricular tachycardia induced by dofetilide (but not by verapamil or ranolazine) at 5× Cmax may have potential as an *in silico* biomarker for screening of the TdP risks posed by candidate molecules.

Although validation is needed to improve the predictive capability of the model, this study demonstrated the possibility of the model to become an effective biomarker to examine the effects of drugs on body-surface ECG parameters using realistic 3D models of the ventricles and torso. This step could lead to our ultimate goal of creating a comprehensive *in silico* drug-safety testing system.

This study also has several limitations. First, in the human ventricular model, we had difficulty in defining the spatial distribution of the sandwiched midcardial cell layer between the endocardial and epicardial cells. The distribution was adopted from the scheme presented in Trudel et al. (2004). Second, in the model of the ventricles and torso, we did not consider the lungs and other tissues between the body surface and the heart when solving for the body surface potentials. The bone located between the heart and body surface might influence ECG data due to its much higher electrical impedance than that of body fluids. We did not consider the effect of this bone in the ECG algorithm. In a future study, the electrical impedance of bone will be considered. Similarly, the effects of non-homogeneous properties of extracellular tissue should be incorporated into the heart model. However, we believe that these limitations did not greatly affect the major findings of this study.

## Data Availability

The datasets generated for this study are available on request to the corresponding author.

## Author CONTRIBUTIONS

D-SY and ES provided the main idea for this research. MH, SH, and MP obtained and analyzed the data. MH wrote the initial draft of the manuscript. D-SY and ES edited the manuscript. CL provided advice on physiological issues and contributed to the toxicity test protocol. All authors reviewed the manuscript.

## Conflict of Interest Statement

MH is employed by SiliconSapiens Inc. (Seoul, South Korea). The remaining authors declare that the research was conducted in the absence of any commercial or financial relationships that could be construed as a potential conflict of interest.

## Abbreviations

AP: action potential
CT: computed tomography
EAD: early afterdepolarization
ECG: electrocardiogram
ORd: O’Hara-Rudy dynamic
TdP: Torsades de pointes
TDR: transmural dispersion of repolarization.
