# Supplementary material for: Three-Dimensional Heart Model-Based Screening of Proarrhythmic Potential by in silico Simulation of Action Potential and Electrocardiograms
Source: Front Physiol. 2019 Sep 4;10:1139. doi: 10.3389/fphys.2019.01139 (PMC6738014; doi:10.3389/fphys.2019.01139)
Supplement: Supplementary file 2 [file Table_2.DOCX]

**Supplementary Figures**


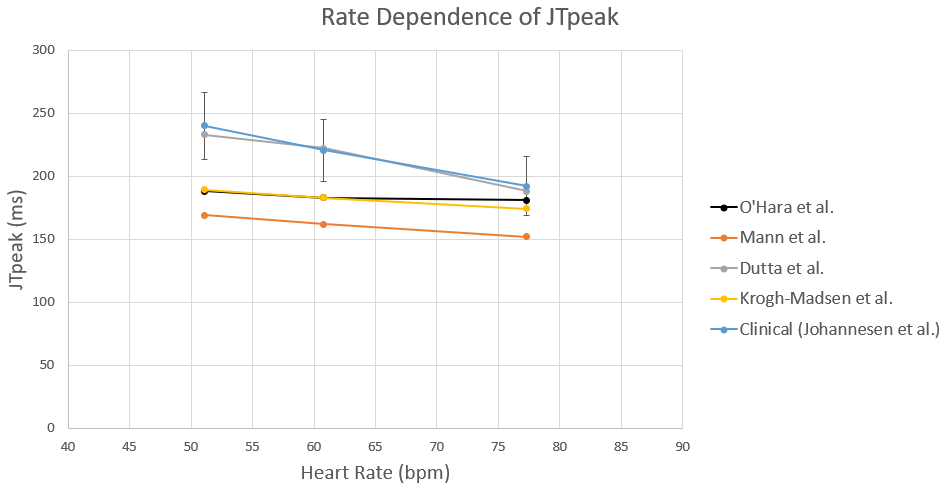


**Fig. S1**. Rate dependence of JTpeak. JTpeak was obtained for different heart rates with different cell models. Simulation results were compared with clinical data by Johannesen et al. (2014)


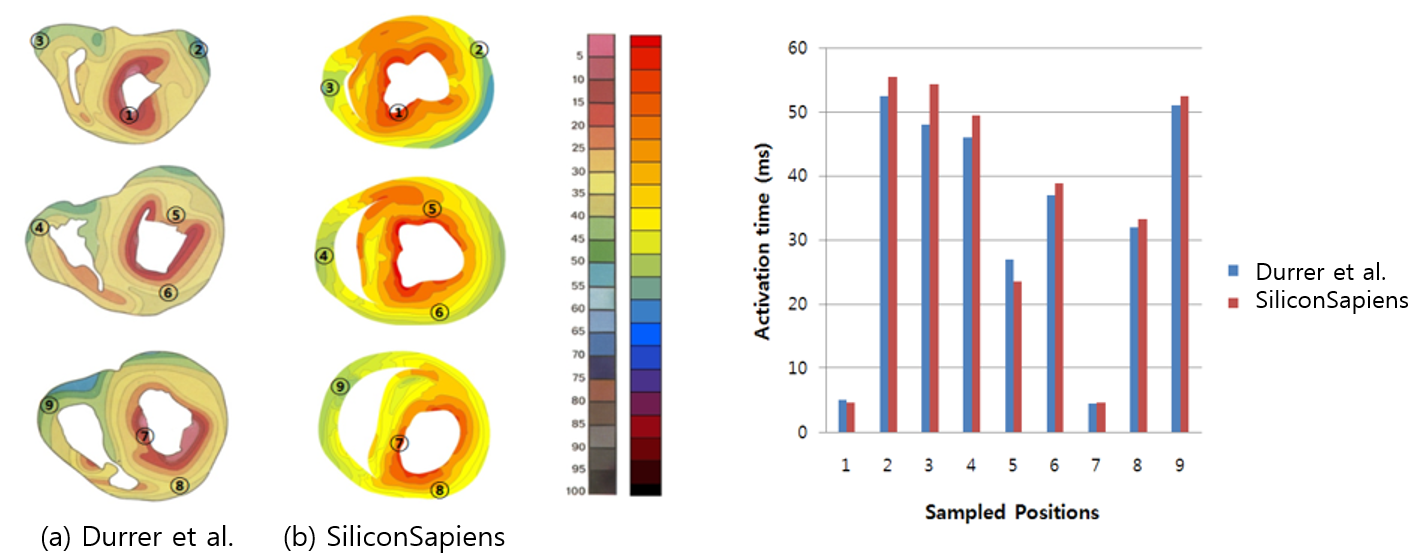


**Fig. S2**. Comparison of activation times in the ventricular model. The activation times obtained from the current ventricular model were compared with those in the literature (Durrer et al., 1970).

**References**

Durrer, D., van Dam, R.T., Freud, G.E., Janse, M.J., Meijler, F.L., Arzbaecher, R.C. (1970), Total excitation of the isolated human heart. Circulation 41, 899-912.

Johannesen, L., Vicente, J., Gray, R.A., Galeotti, L., Loring, Z., Garnett, C.E., Florian, J., Ugander, M., Stockbridge, N., Strauss, D.G. (2014). Improving the assessment of heart toxicity for all new drugs through translational regulatory science. Clinical Pharmacology & Therapeutics 95, 501-508.
